# Predictors of VILI risk: driving pressure, 4DPRR and mechanical power ratio—an experimental study

**DOI:** 10.1186/s40635-024-00697-6

**Published:** 2024-12-11

**Authors:** Mauro Galizia, Valentina Ghidoni, Giulia Catozzi, Stefano Giovanazzi, Domenico Nocera, Beatrice Donati, Tommaso Pozzi, Rosanna D’Albo, Mattia Busana, Federica Romitti, Peter Herrmann, Onnen Moerer, Konrad Meissner, Michael Quintel, Luigi Camporota, Luciano Gattinoni

**Affiliations:** 1 Department of Anaesthesiology, University Medical Center Göttingen, Robert-Koch-Straβe 40, 37075 Göttingen, Germany; 2 Department of Health Sciences, University of Milan, Via Festa del Perdono 7, 20122 Milan, Italia; 3 Department of Health Science, Department of Anesthesia and Intensive Care, AOU Careggi, Largo Brambilla 3, 50139 Florence, Italia; 4 Department of Medical and Surgical Specialties, Radiological Sciences and Public Health, University of Brescia, Piazzale Spedali Civili 1, 25121 Brescia, Italia; 5 Department of Medical and Surgical Sciences, Alma Mater Studiorum, University of Bologna, Via Massarenti 9, 40138 Bologna, Italia; 6 Department of Adult Critical Care, Guy’s and St. Thomas’ NHS Foundation Trust, Health Centre for Human and Applied Physiological Sciences, London, UK

**Keywords:** Ventilator-induced lung injury, Mechanical power, ARDS, Driving pressure, Mechanical ventilation

## Abstract

**Background:**

Ventilator-induced lung injury (VILI) is one of the side effects of mechanical ventilation during ARDS; a prerequisite for averting it is the quantification of its risk factors associated with a given ventilatory setting. Many clinical variables have been proposed as predictors of VILI, of which driving pressure is the most widely used. In this study, we compared the performance of driving pressure, four times the driving pressure added to respiratory rate (4DPRR) and mechanical power ratio.

**Results:**

In a study population of 121 previously healthy pigs exposed to harmful ventilation, we compared the association of driving pressure, 4DPRR and mechanical power ratio to lung weight, lung wet-to-dry and total histological score. All the three variables were associated with these outcomes. Driving pressure, 4DPRR and mechanical power ratio increase linearly with the lung weight (adjusted *R*^2^ of 0.27, 0.36 and 0.40, respectively), the lung wet-to-dry ratio (adjusted *R*^2^ of 0.19, 0.25 and 0.37) and the total histological score (adjusted *R*^2^ of 0.26, 0.38 and 0.26). Using a multiple linear regression model with forward analysis, starting with tidal volume and progressively adding respiratory rate and positive end-expiratory pressure, and comparing the topic with the outcome variables, we obtained R^2^ values, respectively, of 0.07, 0.20, 0.42 for lung weight, 0.09, 0.19, 0.26 for lung wet-to-dry ratio and 0.07, 0.27, 0.43 for total histological score.

**Conclusions:**

Driving pressure, 4DPRR and mechanical power ratio, were all associated with lung injury in healthy animals undergoing mechanical ventilation.

## Background

The term ventilator or ventilation-induced lung injury (VILI) refers to the direct lung damage caused by ventilation, whether mechanical or spontaneous. Over the years, the emphasis has been placed on the link between VILI and excessive pressure (i.e. barotrauma [1]), excessive volume (i.e. volutrauma [2]), excessive alveolar instability and heterogeneity leading to regional repeated alveolar open and closing (i.e. atelectrauma [3]) and, more recently, to the excessive total mechanical energy delivered per unit of time (i.e. ergotrauma [4]). These mechanical forces will also induce biotrauma as mechanical stimuli are converted into biological inflammatory reactions in a process of mechanotransduction [5]. It should be realised, however, that although the focus of VILI has been centred on single causative triggers, VILI is likely to result from the interplay of different triggers which may act simultaneously.

The identification and quantification of the risk factors for VILI is of major importance for the application of respiratory support in critically ill patients. In this study, we tested three clinical predictor variables of VILI. The first, driving pressure [6], is the most widely used in clinical practice. The second variable include respiratory rate and driving pressure and is calculated as the addition of respiratory rate to four times the driving pressure (4DPRR) [7]. This variable has been shown to be associated to the risk of death in an analysis of trial data. A third variable is the mechanical power ratio [8] (i.e. actual energy load to the respiratory system relative to the expected energy load in normal healthy subjects) which in addition to driving pressure and respiratory rate, includes also positive end-expiratory pressure (PEEP). Although the addition of PEEP to the mechanical power is still controversial given that PEEP is considered a static and not a dynamic variable and therefore should not be multiplied by the respiratory rate, experimental study supports the inclusion of PEEP in the mechanical power equation [9].

In this retrospective study, which includes 121 pigs treated for 48 h with a different ventilatory settings, we aimed to compare the extent to which the three variables—driving pressure, 4DPRR and mechanical power ratio﻿—, were associated with VILI, as measured by lung weight, lung wet-to-dry ratio and total histological score. The hypothesis was that the mechanical power ratio would show a stronger association with injury markers, as it encompasses all ventilator variables linked to VILI.

## Methods

### Study population

The study population consisted of 121 healthy female pigs included in previous experiments on VILI [10–12]. A detailed explanation of the ventilatory settings for each experiment is provided in Table 1.

**Table 1:** Study population formation

Experiment	Definition of groups					Number of pigs
		MP, J⋅min^−1^	Vt, ml⋅kg^−1^	RR, breaths⋅min^−1^	PEEP, cmH_2_O	
Vassalli et al. Anesthesiology. 2020 [10]	Low MP	15	30–35^*^	7–15	5	42 pigs, 7 equally distributed in each group
			10–14	40	5	
			10–14	11–21	25	
	High MP	30	30–35^*^	7–15	5	
			10–14	40	5	
			10–14	11–21	25	
Romitti et al. Physiological Reports. 2022 [11]	Low MP	3	6.9 ± 0.3	15.7 ± 1.0	4	18 pigs, 6 equally distributed in each group
	Mid-MP	7	9.7 ± 0.7	20.7 ± 1.4	4	
	High MP	12	11.5 ± 1.1	23.3 ± 1.5	4	
Busana et al. Journal of applied physiology. 2022 [12]	Passive expiratory flow	8–9	20	9	6	22 pigs, 11 equally distributed in each group
	Controlled expiratory flow					
Gattarello et al. Under review. 2024	Low MP	6.2	9.7	15	7	39 pigs, 19 in low MP group and 20 in high MP group
	High MP	18.1	20	15	7	

The experiments started at the baseline with identical ventilatory settings for the diverse study groups, employing volume-controlled ventilation. Subsequently, the ventilation was modified in accordance with the specific experimental design to achieve the desired target. In the first experiment [10], two distinct MP groups were delineated (low MP groups, approaching 15 J⋅min^−1^, and high MP groups, approximating 30 J⋅min^−1^). The different MP values were obtained by varying Vt, RR, or PEEP exclusively and maintaining the other two parameters constant throughout the experiment. In the second experiment [11] three groups were identified through MP values (respectively, 3, 7 and 12 J⋅min^−1^) by varying both tidal volume and respiratory rate. The third experiment [12] comprised two distinct groups obtained by varying the expiratory time through a valve which modified the resistive component of the mechanical power, while the MP target was identical for both groups (8–9 J⋅min^−1^). The fourth experiment included of two different groups with low and high MP (6.2 J⋅min^−1^ and 18.1 J⋅min^−1^)

^*^Vt has been set to reach strain of 2.5, after measuring functional residual capacity with helium dilution method at baseline

### Experimental protocol

The following variables were collected in each experiment at baseline, at 0.5 h after protocol initiation, and at 6 h intervals until the 48th hour. For each variable we calculated the average over the experimental period, excluding the baseline measurement, with the exception of the outcome variables, which were collected at the end of the experiment.

### Respiratory mechanics variables

The respiratory mechanics variables were computed as follows:

$$\text{Respiratory system elastance }\left({\text{E}}_{\text{rs}}, {\text{cmH}}_{2}\text{O}\cdot {\text{L}}^{-1}\right)=\frac{{\text{Paw}}_{\text{plat}}-\text{PEEP}}{\text{Vt}},$$

$$\text{Chest wall elastance} {(\text{E}}_{\text{cw}}, {\text{cmH}}_{2}\text{O}\cdot {\text{L}}^{-1})=\frac{{\text{Pes}}_{\text{plat}}-{\text{Pes}}_{\text{ee}}}{\text{Vt}},$$

$$\text{Lung elastance} \left(\text{EL }, {\text{cmH}}_{2}\text{O}\cdot {\text{L}}^{-1}\right)=\text{ Ers}-\text{ Ecw},$$

where Paw_plat_ is plateau airway pressure, PEEP is the end-expiratory airway pressure, Pes_plat_ is esophageal pressure measured at inspiratory plateau pause, Pes_ee_ is end-expiratory esophageal pressure measured at PEEP. Pressures are expressed in cmH_2_O, and tidal volume (Vt) in litres.

$$\text{Respiratory system compliance} \left({\text{C}}_{\text{rs}},\text{ ml}\cdot {\text{cmH}}_{2}{\text{O}}^{-1}\right)=\frac{\text{Vt}}{{\text{Paw}}_{\text{plat}}-\text{PEEP}},$$

where Vt is the tidal volume (ml) and the pressures at the denominator are the same explained in the elastance formulas.

### Anatomopathological findings and histological score

Autopsy was performed immediately after euthanasia. Lung weight was measured immediately, and six samples were collected for the lung wet-to-dry measurement. End-experiment lung weight and lung wet-to-dry ratio were used as primary outcome given that they represent and reflect the degree of lung damage induced by the ventilation. The lung wet-to-dry ratio was calculated as the ratio between the weight of the sample and its weight after 24 h of drying at 50 Celsius degrees in an oven. Ten tissue samples were obtained from each lung, equally distributed, for histological analysis. For each sample, the presence of given alterations (i.e. alveolar rupture, inflammation, alveolar oedema, and atelectasis) was expressed according to the prevalence at which the alteration was observed in the field and a score was assigned (prevalence 0–0.25, score 2; 0.25–0.50, score 4; 0.50–0.75, score 8; 0.75–1.00, score 16). The total histological score was then calculated as the sum of every singular score.

### Predictive variables

Driving pressure was computed as:

$$\text{DP}=\frac{\text{Vt}}{{\text{C}}_{\text{rs}}},$$

where DP is driving pressure (cmH_2_O), Vt is tidal volume (ml), C_rs_ is the compliance of the respiratory system (ml · cmH_2_O^−1^).

4DPRR was computed as:

$$4\text{DPRR}=4\times \text{DP}+\text{RR},$$

where DP is driving pressure (cmH_2_O), RR is the respiratory rate (breaths · min^−1^).

Mechanical power was computed as:

$$\text{MP}=0.098\times \text{RR}\times \{{{\text{V}}_{\text{T}}}^{2}\times \left[\frac{1}{2}\times {\text{E}}_{\text{rs}}+\text{RR}\times \frac{1+\text{I}:\text{E}}{60\times \text{I}:\text{E}}\times {\text{R}}_{\text{aw}}\right]+{\text{V}}_{\text{T}}\times \text{PEEP}\},$$

where RR is respiratory rate (breaths · min^−1^), Vt is the tidal volume (litres), E_rs_ is the elastance of the respiratory system (cmH_2_O · l^−1^), R_aw_ is the resistance of the airways (cmH_2_O · l^−1^), PEEP is the positive end-expiratory pressure (cmH_2_O).

Note that driving pressure is present in all the three variables (Vt times elastance equals Vt · C_rs_^−1^, i.e. driving pressure), the respiratory rate is present into 4DPRR and mechanical power; PEEP times Vt is present exclusively into mechanical power.

Mechanical power ratio was computed as:

$$\text{Mechanical power ratio}=\frac{\text{Actual mechanical power}}{\text{Expected mechanical power}},$$

where the expected mechanical power is computed using the normal physiological values of healthy adult pigs in according to De Robertis [13]: RR of 20 breaths · min^−1^, Vt of 10 ml · kg^−1^, E_rs_ of 0.75 cmH_2_O · l^−1^ · kg^−1^, I:E of 0.5, R_aw_ of 3.9 cmH_2_O · l^−1^ · kg^−1^, PEEP = 0 cmH_2_O.

### Statistical analysis

Continuous variables are presented as mean and standard deviation. A linear regression model was employed to assess the relationship between continuous variables (predictive variables as a function of outcome variables). A multiple linear regression model, with a forward method, was used to assess the relationship between the constitutive variables of the predictive ones (i.e. tidal volume, respiratory rate and positive end-expiratory pressure) against the outcome variables. For statistical analysis R 4.3.1 (R Studio, ver. 2023.09.1 + 494, by Posit Software) was used.

## Results

**Fig. 1:**
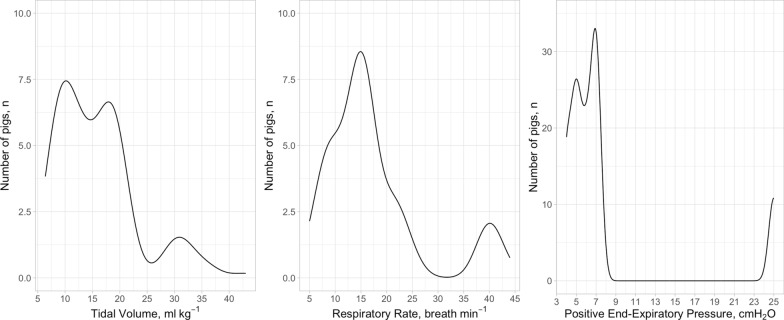
Distribution of the main determinants of the predictive variables in the study population. Distribution of tidal volume, respiratory rate and positive end-expiratory pressure in the whole study population

Under general anaesthesia, all animals were ventilated with driving pressure ranging from 5.0 to 48.8 cmH_2_O, 4DPRR ranging from 35 to 266, and mechanical power ratio ranging from 0.86 to 26.4. In Table 2, we summarised the main respiratory mechanical characteristics of the study population. The distribution of tidal volume, respiratory rate and PEEP, the key variables which determine driving pressure, 4DPRR and mechanical power ratio are presented in Fig. 1. As shown, while the tidal volume and respiratory rate had a wide distribution, the PEEP values used in this population had a narrower distribution range.

**Table 2:** Average applied respiratory variables

Number of pigs and weight	n = 121, w = 27.7 ± 3.66
Driving pressure, *cmH*_*2*_*O*	14.90 ± 6.64 (5.67, 39.40)
Driving pressure times 4 + respiratory rate	77.0 ± 26.8 (37.7, 162.0)
Mechanical power, *Joules⋅ min*^*−1*^	13.80 ± 8.67 (2.95, 57.60)
Mechanical power ratio	5.56 ± 3.99 (0.95, 23.90)
Positive end-expiratory pressure, *cmH*_*2*_*O*	7.96 ± 6.28 (4.00, 25.00)
Tidal volume, *ml⋅ kg*^*−1*^	15.90 ± 7.41 (6.39, 43.00)
Respiratory rate, *breaths⋅min*^*−1*^	17.30 ± 9.49 (5.00, 44.00)
Minute ventilation, *litres⋅ min*^*−1*^	6.66 ± 3.04 (2.86, 15.30)

The data are presented as mean ± SD (range). Each variable is computed as the mean over the experimental period

**Fig. 2:**
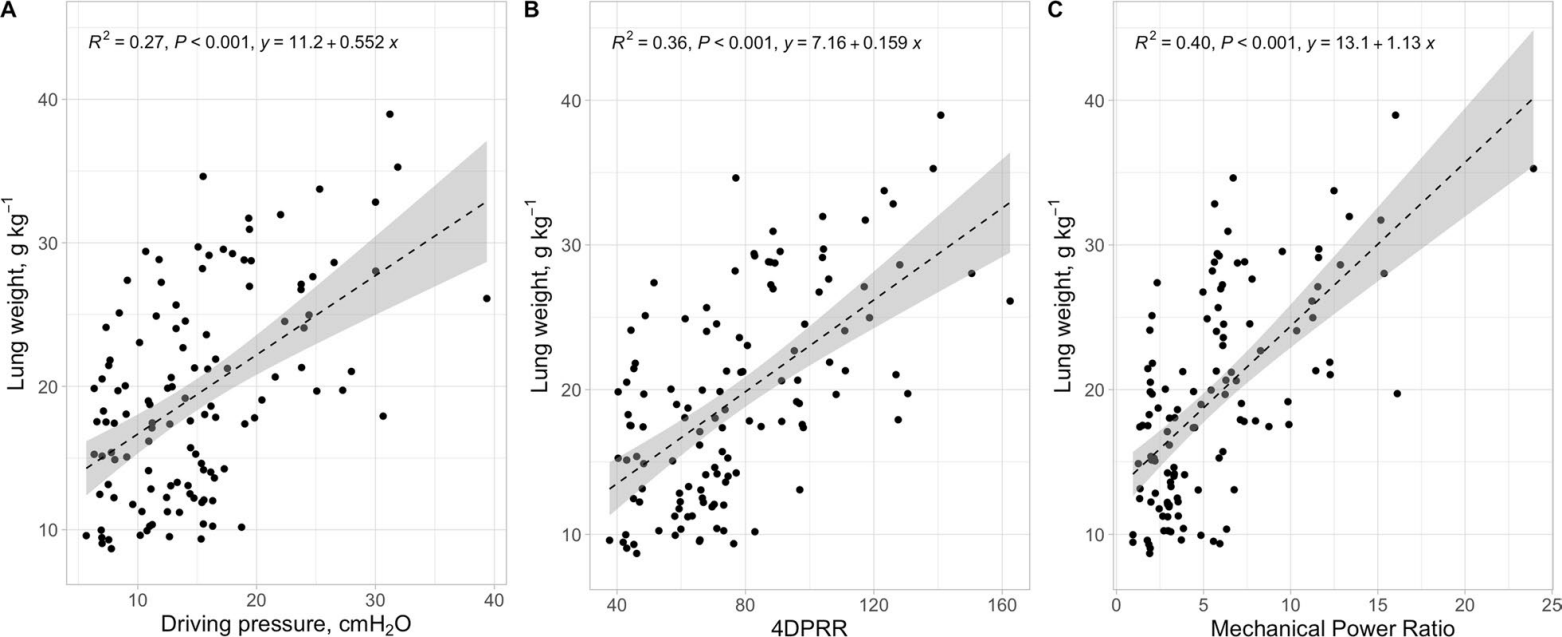
Lung weight–predictive variables relationship. Lung weight as a function of driving pressure (**A**), 4DPRR (**B**) and mechanical power ratio (**C**). R^2^ of 0.27, 0.36 and 0.40, respectively

**Fig. 3:**
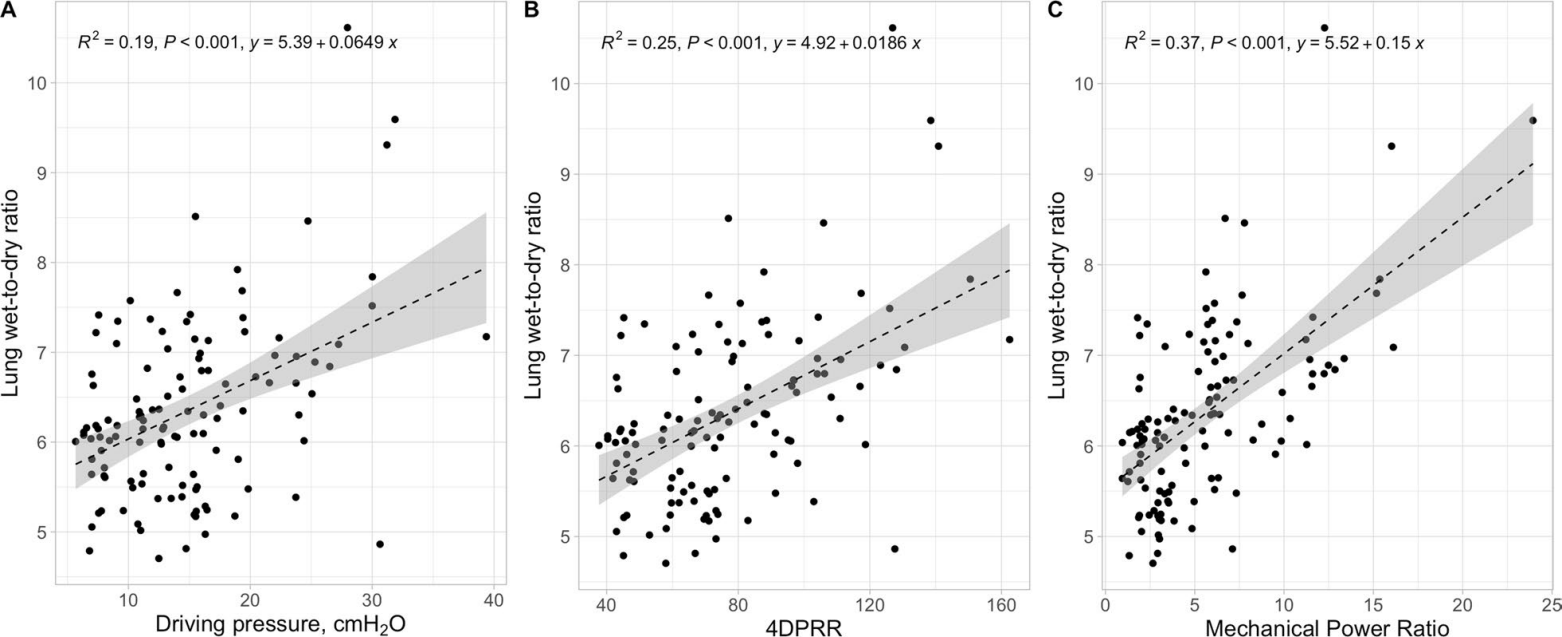
Lung wet-to-dry–predictive variables relationship. Lung wet-to-dry as a function of driving pressure (**A**), 4DPRR (**B**) and mechanical power ratio (**C**). R^2^ of 0.19, 0.25 and 0.37, respectively

**Fig. 4:**
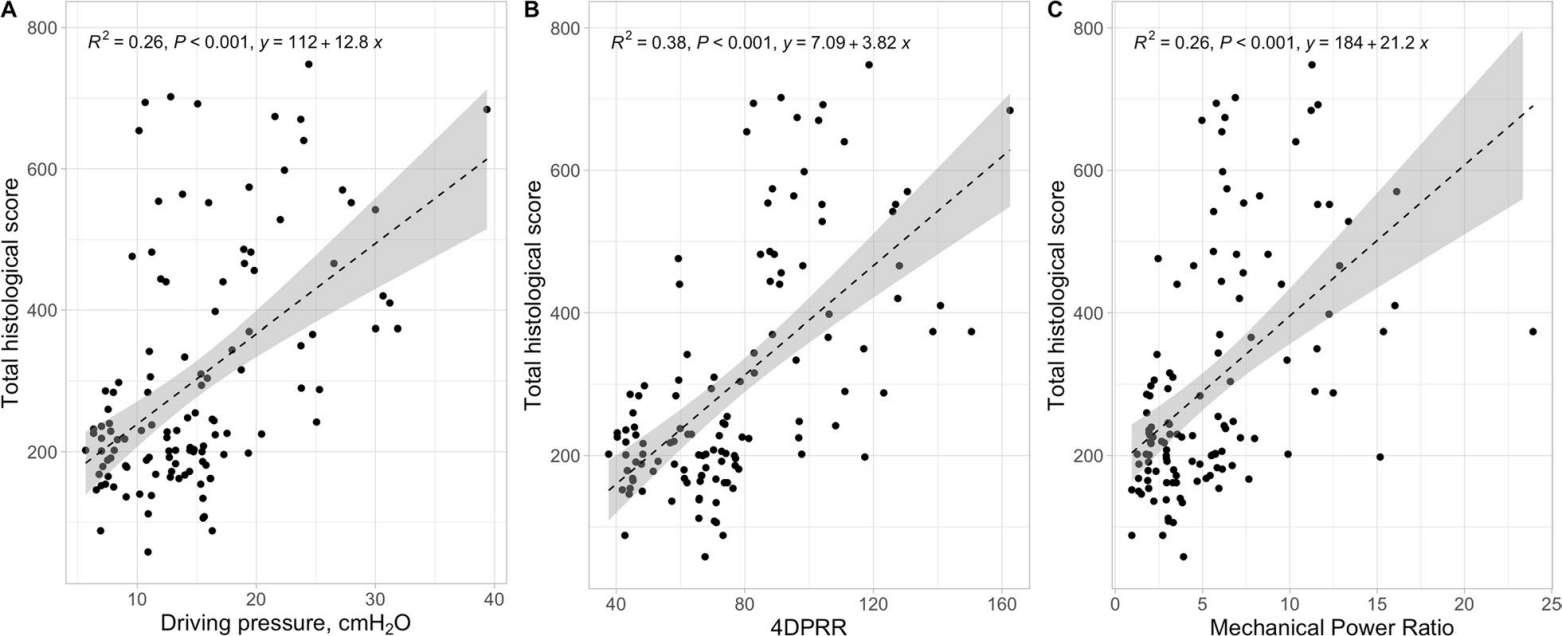
Total histological score–predictive variables relationship. Total histological score as a function of driving pressure (**A**), 4DPRR (**B**) and mechanical power ratio (**C**). R^2^ of 0.26, 0.38 and 0.26, respectively

In Fig. 2, we present the relationship between lung weight, an indicator of lung oedema, as a function of driving pressure, 4DPRR and mechanical power ratio. As shown, although all the three predictive variables were significantly related to the lung weight, the adjusted R^2^ was 0.27, for driving pressure, to 0.36, for 4DPRR, and 0.40 for the mechanical power ratio. A similar behaviour was found when considering the lung wet-to-dry ratio (adjusted R^2^ increased from 0.19 to 0.25 and 0.37), and total histological score (adjusted R^2^ from 0.26 to 0.38 and 0.26), as shown in Figs. 3 and 4.

Table 3 shows the correlation between key ventilation variables (i.e. tidal volume, respiratory rate and PEEP) and outcome variables (lung weight, lung wet-to-dry ratio and total histological score). Using a forward approach, we first compared tidal volume with the three outcome variables, obtaining an adjusted R2 of 0.07, 0.09 and 0.07, respectively. Adding first the respiratory rate and then PEEP, we obtained an increase in correlation up to an adjusted R2 of 0.42, 0.26 and 0.43. Mathematically, the use of the tidal volume reflects the driving pressure to which, the addition of the respiratory rate is captures by the 4DPRR and, finally, the addition of the PEEP is reflected in the mechanical power ratio.

**Table 3:** Adjusted R^2^ of predictive variables determinants and outcomes

	Tidal volume, *ml⋅ kg*^*−1*^+	Respiratory rate, *breaths⋅min*^*−1*^+	PEEP, *cmH*_*2*_*O*
Lung weight, *g⋅ kg*^*−1*^			
Adjusted R^2^	0.07	0.20	0.42
p-value	0.002	< 0.001	< 0.001
Lung wet-to-dry ratio			
Adjusted R^2^	0.09	0.19	0.26
p-value	< 0.001	< 0.001	< 0.001
Total histological score			
Adjusted R^2^	0.07	0.27	0.43
p-value	0.002	< 0.001	< 0.001

Tidal volume is the main determinant of driving pressure, tidal volume + respiratory rate are the main determinants of 4DPRR, tidal volume + respiratory rate + PEEP are the main determinants of mechanical power ratio. The multiple linear regression model, utilising a forward approach, reports the adjusted R-squared value obtained by first incorporating the respiratory rate and then the PEEP into the tidal volume, with the outcomes employed as dependent variables

## Discussion

The main result of the study was that in the studied population, driving pressure, 4DPRR, and mechanical power ratio were all significantly and similarly associated with VILI, as assessed by lung weight, lung wet-to-dry ratio, and histology. Driving pressure is determined by tidal volume and respiratory system elastance, 4DPRR is determined by tidal volume, respiratory system elastance and respiratory rate, while mechanical power is determined by tidal volume, respiratory system elastance, respiratory rate and PEEP. Each of these determinants, taken individually, has been shown to produce VILI [9, 14–18]. The novelty of this study lies in its approach: unlike pathological studies where disease effects can obscure the causal impact of ventilation on VILI outcomes, we begin with a healthy lung model, enabling us to gather anatomical and histological data. This approach allows us to explore a range of driving pressure and mechanical power ratio that have not yet been tested in clinical settings. The above-mentioned predictive variables are commonly used clinically or experimentally to ensure the safety of mechanical ventilation although each of them has its own origins and limitations.

### Driving pressure

The association of this variable with outcome was described in 2015 [6] after a retrospective analysis of ten randomised trials. Four of them [19–22] were used to build the model (336 patients). The ARMA trial [14] was used as a first validation cohort (861 patients) and the other five studies [23–27] (2365 patients) were used as the second validation cohort. The analysis concluded that driving pressure was a better predictor of outcome than tidal volume or PEEP. Conceptually, driving pressure is the tidal volume normalised to the compliance of the patient’s lungs which is related to the lung gas content [28]. Therefore, compared to tidal volume per kg, driving pressure better reflects the strain on the ventilatable part of the lung, a well-recognised component of VILI.

As shown in an experimental study where we monitored the development of VILI using CT scans, we observed that the same driving pressure (or injurious tidal volume) delivered at a rate of six breaths per minute for 48 h did not cause lung lesions, whereas doubling the frequency to 12 breaths per minute resulted in notable lung damage [16]. Regarding PEEP, on the other hand, there are two effects to consider: the first is its contribution to the total stress to which the extracellular matrix of the lung is exposed; the second, is its contribution to the strain [29].

### 4DPRR

**Fig. 5:**
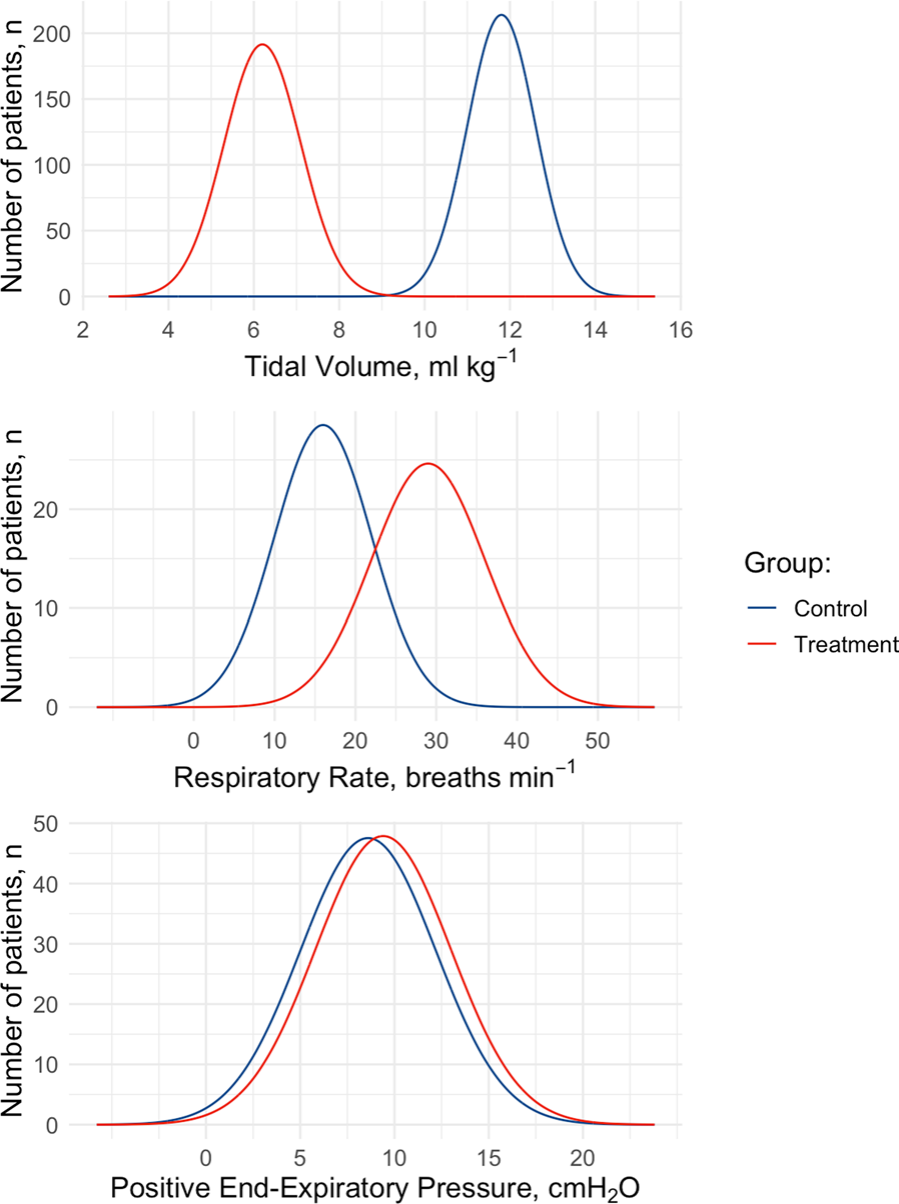
Theoretical distribution of the main determinants of the predictive variables in the ARMA study population. Distribution of tidal volume, respiratory rate and positive end-expiratory pressure in the ARMA study population [14], assuming a normal distribution of the variables

The advantage of 4DPRR is the inclusion of the respiratory rate, being calculated as driving pressure times four plus respiratory rate [7]. This variable provided similar results to mechanical power in outcome prediction. The potential disadvantage of 4DPRR is that it is based on epidemiological models and may be population-specific [30]. In Fig. 5, we report, as an example, the population characteristics of the ARMA trial [14] which was used as a validation cohort number 1 in determining driving pressure and one of the major contributors of 4DPRR determination. As shown, tidal volume and respiratory rate were differently distributed in the arms of the study, while the PEEP was identical. Therefore, the potential value of PEEP in this population is not explored. Finally, although it is possible that “high PEEP” may also increase the driving pressure and 4DPRR, depending on tidal volume, the precise quantification of the effect of a specific level of PEEP on VILI cannot be tested using these methods unless it affects driving pressure.

### Mechanical power

Mechanical power incorporates driving pressure, respiratory rate, and PEEP, theoretically making it a more comprehensive predictor of VILI. By considering all components—tidal volume, respiratory rate, and PEEP—it could provide additional insights to optimise mechanical ventilation. However, the results do not demonstrate a distinct advantage of mechanical power ratio over driving pressure and 4DPRR. This finding may suggest several possibilities: (1) the impact of increased PEEP may be largely reflected in driving pressure changes at higher lung volumes, with driving pressure capturing most of the effects of PEEP; (2) PEEP may play a less significant role in this model of initially healthy lungs; and (3) the variations of PEEP values within the ranges of tested mechanical power ratio may be narrower than the variations of driving pressure or respiratory rate, with PEEP ultimately having minimal additional impact compared to driving pressure or 4DPRR.

### Study limitations

This study presents several potential limitations that may impact the interpretation and applicability of its findings. Firstly, the use of a porcine model offers valuable insights into VILI mechanisms; however, these findings cannot be directly applied to human clinical settings. The porcine models, while informative, do not fully capture the complex dynamics of human ARDS, particularly as they start with healthy lungs and do not account for patient-specific factors and comorbidities. Secondly, the study relies on a limited set of markers (lung weight, lung wet-to-dry ratio, and histological score) to assess lung injury. These markers may not capture all the facets of VILI, especially those affecting longer-term functional outcomes and the potential for lung recovery. Thirdly, while the data are derived from animals across several different experiments that test various aspects of VILI, the heterogeneity of these experiments does not constitute a single uniform experimental plan. This might affect the uniformity and consistency of the results. Additionally, some of the epidemiological data used may not align well with experimental models of ARDS, which complicates the translation of these findings to human patients. The variability inherent in human clinical practice, where patients' conditions and responses to ventilatory settings can vary significantly, makes it challenging to generalise these results to broader clinical scenarios without further validation in human trials. Nevertheless, this study does provide additional data at a biological, physiological and anatomical level on the effect of ventilation variables such as driving pressure, 4DPRR and mechanical power ration on the macroscopic and pathological markers of VILI.

## Conclusions

Driving pressure, 4DPRR and mechanical power ratio, were all associated with lung injury in healthy animals undergoing mechanical ventilation.

## Data Availability

The datasets used and/or analysed during the current study are available from the corresponding authors on reasonable request. This article has no online data supplement.

## Abbreviations

VILI: Ventilator-induced lung injury
DP: Driving pressure
4DPRR: Driving pressure times four plus respiratory rate
MP: Mechanical power
ARDS: Acute respiratory distress syndrome
Ers: Respiratory system elastance
Ecw: Chest wall elastance
EL: Lung elastance
Crs: Respiratory system elastance
PEEP: Positive end-expiratory pressure
FRC: Functional residual capacity
Vt: Tidal volume
Raw: Airways resistance
RR: Respiratory rate
I:E: Inspiratory-to-expiratory time ratio

## References

[1] Kumar A, Pontoppidan H, Falke KJ, Wilson RS, Laver MB (1973) Pulmonary barotrauma during mechanical ventilation. Crit Care Med 1(4):181–186. 10.1097/00003246-197307000-00001 4587509 10.1097/00003246-197307000-00001

[2] Dreyfuss D, Soler P, Basset G, Saumon G (1988) High inflation pressure pulmonary edema. Respective effects of high airway pressure, high tidal volume, and positive end-expiratory pressure. Am Rev Respir Dis 137(5):1159–1164. 10.1164/ajrccm/137.5.1159 3057957 10.1164/ajrccm/137.5.1159

[3] Tremblay L, Valenza F, Ribeiro SP, Li J, Slutsky AS (1997) Injurious ventilatory strategies increase cytokines and c-fos m-RNA expression in an isolated rat lung model. J Clin Invest 99(5):944–952. 10.1172/JCI119259 9062352 10.1172/JCI119259 PMC507902

[4] Marini JJ (2018) Dissipation of energy during the respiratory cycle: conditional importance of ergotrauma to structural lung damage. Curr Opin Crit Care 24(1):16–22. 10.1097/MCC.0000000000000470 29176330 10.1097/MCC.0000000000000470

[5] Du H et al (2023) Tuning immunity through tissue mechanotransduction. Nat Rev Immunol 23(3):174–188. 10.1038/s41577-022-00761-w 35974148 10.1038/s41577-022-00761-w PMC9379893

[6] Amato MBP et al (2015) Driving pressure and survival in the acute respiratory distress syndrome. N Engl J Med 372(8):747–755. 10.1056/NEJMsa1410639 25693014 10.1056/NEJMsa1410639

[7] Costa ELV et al (2021) Ventilatory variables and mechanical power in patients with acute respiratory distress syndrome. Am J Respir Crit Care Med 204(3):303–311. 10.1164/rccm.202009-3467OC 33784486 10.1164/rccm.202009-3467OC

[8] Dlbo R et al (2024) Mechanical power ratio threshold for ventilator-induced lung injury. Intensive Care Med Exp 12(1):65. 10.1186/s40635-024-00649-0 39080225 10.1186/s40635-024-00649-0 PMC11289208

[9] Collino F et al (2019) Positive end-expiratory pressure and mechanical power. Anesthesiology 130(1):119–130. 10.1097/ALN.0000000000002458 30277932 10.1097/ALN.0000000000002458

[10] Vassalli F et al (2020) Does iso-mechanical power lead to iso-lung damage?: An experimental study in a porcine model. Anesthesiology 132(5):1126–1137. 10.1097/ALN.0000000000003189 32032095 10.1097/ALN.0000000000003189

[11] Romitti F et al (2022) Mechanical power thresholds during mechanical ventilation: An experimental study. Physiol Rep 10(6):e15225. 10.14814/phy2.15225 35340133 10.14814/phy2.15225 PMC8957661

[12] Busana M et al (2022) Energy dissipation during expiration and ventilator-induced lung injury: an experimental animal study. J Appl Physiol 133(5):1212–1219. 10.1152/japplphysiol.00426.2022 36173324 10.1152/japplphysiol.00426.2022

[13] De Robertis E, Liu JM, Blomquist S, Dahm PL, Thörne J, Jonson B (2001) Elastic properties of the lung and the chest wall in young and adult healthy pigs. Eur Respir J 17(4):703–711. 10.1183/09031936.01.17407030 11401067 10.1183/09031936.01.17407030

[14] Acute Respiratory Distress Syndrome Network (2000) Ventilation with lower tidal volumes as compared with traditional tidal volumes for acute lung injury and the acute respiratory distress syndrome. N Engl J Med 342(18):1301–1308. 10.1056/NEJM200005043421801 10793162 10.1056/NEJM200005043421801

[15] Protti A, Votta E, Gattinoni L (2014) Which is the most important strain in the pathogenesis of ventilator-induced lung injury: dynamic or static? Curr Opin Crit Care 20(1):33–38. 10.1097/MCC.0000000000000047 24247615 10.1097/MCC.0000000000000047

[16] Cressoni M et al (2016) Mechanical power and development of ventilator-induced lung injury. Anesthesiology 124(5):1100–1108. 10.1097/ALN.0000000000001056 26872367 10.1097/ALN.0000000000001056

[17] Protti A et al (2016) Role of strain rate in the pathogenesis of ventilator-induced lung edema. Crit Care Med 44(9):e838–e845. 10.1097/CCM.0000000000001718 27054894 10.1097/CCM.0000000000001718

[18] Cavalcanti AB et al (2017) Effect of lung recruitment and titrated positive end-expiratory pressure (PEEP) vs low PEEP on mortality in patients with acute respiratory distress syndrome. JAMA 318(14):1335. 10.1001/jama.2017.14171 28973363 10.1001/jama.2017.14171 PMC5710484

[19] Amato MB et al (1998) Effect of a protective-ventilation strategy on mortality in the acute respiratory distress syndrome. N Engl J Med 338(6):347–354. 10.1056/NEJM199802053380602 9449727 10.1056/NEJM199802053380602

[20] Brochard L et al (1998) Tidal volume reduction for prevention of ventilator-induced lung injury in acute respiratory distress syndrome. The Multicenter Trail Group on Tidal Volume reduction in ARDS. Am J Respir Crit Care Med 158(6):1831–1838. 10.1164/ajrccm.158.6.9801044 9847275 10.1164/ajrccm.158.6.9801044

[21] Stewart TE et al (1998) Evaluation of a ventilation strategy to prevent barotrauma in patients at high risk for acute respiratory distress syndrome. Pressure- and volume-limited ventilation strategy group. N Engl J Med 338(6):355–361. 10.1056/NEJM199802053380603 9449728 10.1056/NEJM199802053380603

[22] Brower RG et al (1999) Prospective, randomized, controlled clinical trial comparing traditional versus reduced tidal volume ventilation in acute respiratory distress syndrome patients. Crit Care Med 27(8):1492–1498. 10.1097/00003246-199908000-00015 10470755 10.1097/00003246-199908000-00015

[23] Briel M et al (2010) Higher vs lower positive end-expiratory pressure in patients with acute lung injury and acute respiratory distress syndrome: systematic review and meta-analysis. JAMA 303(9):865–873. 10.1001/jama.2010.218 20197533 10.1001/jama.2010.218

[24] Brower RG et al (2004) Higher versus lower positive end-expiratory pressures in patients with the acute respiratory distress syndrome. N Engl J Med 351(4):327–336. 10.1056/NEJMoa032193 15269312 10.1056/NEJMoa032193

[25] Mercat A et al (2008) Positive end-expiratory pressure setting in adults with acute lung injury and acute respiratory distress syndrome: a randomized controlled trial. JAMA 299(6):646–655. 10.1001/jama.299.6.646 18270353 10.1001/jama.299.6.646

[26] Meade MO et al (2008) Ventilation strategy using low tidal volumes, recruitment maneuvers, and high positive end-expiratory pressure for acute lung injury and acute respiratory distress syndrome: a randomized controlled trial. JAMA 299(6):637–645. 10.1001/jama.299.6.637 18270352 10.1001/jama.299.6.637

[27] Talmor D et al (2008) Mechanical ventilation guided by esophageal pressure in acute lung injury. N Engl J Med 359(20):2095–2104. 10.1056/NEJMoa0708638 19001507 10.1056/NEJMoa0708638 PMC3969885

[28] Gattinoni L, Pesenti A, Avalli L, Rossi F, Bombino M (1987) Pressure–volume curve of total respiratory system in acute respiratory failure. Computed tomographic scan study. Am Rev Respir Dis 136(3):730–736. 10.1164/ajrccm/136.3.730 3307572 10.1164/ajrccm/136.3.730

[29] Mentzelopoulos SD, Roussos C, Zakynthinos SG (2005) Prone position reduces lung stress and strain in severe acute respiratory distress syndrome. Eur Respir J 25(3):534–544. 10.1183/09031936.05.00105804 15738300 10.1183/09031936.05.00105804

[30] Camporota L, Busana M, Marini JJ, Gattinoni L (2021) The 4DPRR index and mechanical power: a step ahead or four steps backward? Am J Respir Crit Care Med 204(4):491–492. 10.1164/rccm.202104-0923LE 34081879 10.1164/rccm.202104-0923LE PMC8480246

